# Ectopy-triggering ganglionated plexuses ablation to prevent atrial fibrillation: GANGLIA-AF study

**DOI:** 10.1016/j.hrthm.2021.12.010

**Published:** 2022-04

**Authors:** Min-Young Kim, Clare Coyle, David R. Tomlinson, Markus B. Sikkel, Afzal Sohaib, Vishal Luther, Kevin M. Leong, Louisa Malcolme-Lawes, Benjamin Low, Belinda Sandler, Elaine Lim, Michelle Todd, Michael Fudge, Ian J. Wright, Michael Koa-Wing, Fu Siong Ng, Norman A. Qureshi, Zachary I. Whinnett, Nicholas S. Peters, Daniel Newcomb, Cherith Wood, Gurpreet Dhillon, Ross J. Hunter, Phang Boon Lim, Nicholas W.F. Linton, Prapa Kanagaratnam

**Affiliations:** ∗ Myocardial Function Section, National Heart and Lung Institute, Imperial College London, London, United Kingdom; † Cardiology Department, Hammersmith Hospital, Imperial College Healthcare NHS Trust, London, United Kingdom; ‡ Imperial Centre for Cardiac Engineering, Imperial College London, London, United Kingdom; § Cardiology Department, Derriford Hospital, University Hospitals Plymouth NHS Trust, Plymouth, United Kingdom; ‖ Cardiology Department, St Bartholomew’s Hospital, Barts Health NHS Trust, London, United Kingdom; ¶ Department of Bioengineering, Imperial College London, London, United Kingdom

**Keywords:** Paroxysmal atrial fibrillation, Autonomic nervous system, Neuromodulation, Ganglionated plexi, Ganglionated plexuses

## Abstract

**Background:**

The ganglionated plexuses (GPs) of the intrinsic cardiac autonomic system may play a role in atrial fibrillation (AF).

**Objective:**

We hypothesized that ablating the ectopy-triggering GPs (ET-GPs) prevents AF.

**Methods:**

GANGLIA-AF (ClinicalTrials.gov identifier NCT02487654) was a prospective, randomized, controlled, 3-center trial. ET-GPs were mapped using high frequency stimulation, delivered within the atrial refractory period and ablated until nonfunctional. If triggered AF became incessant, atrioventricular dissociating GPs were ablated. We compared GP ablation (GPA) *without* pulmonary vein isolation (PVI) against PVI in patients with paroxysmal AF. Follow-up was for 12 months including 3-monthly 48-hour Holter monitors. The primary end point was documented ≥30 seconds of atrial arrhythmia after a 3-month blanking period.

**Results:**

A total of 102 randomized patients were analyzed on a per-protocol basis after GPA (n = 52; 51%) or PVI (n = 50; 49%). Patients who underwent GPA had 89 ± 26 high frequency stimulation sites tested, identifying a median of 18.5% (interquartile range 16%–21%) of GPs. The radiofrequency ablation time was 22.9 ± 9.8 minutes in GPA and 38 ± 14.4 minutes in PVI (*P* < .0001). The freedom from ≥30 seconds of atrial arrhythmia at 12-month follow-up was 50% (26 of 52) with GPA vs 64% (32 of 50) with PVI (log-rank, *P* = .09). ET-GPA without atrioventricular dissociating GPA achieved 58% (22 of 38) freedom from the primary end point. There was a significantly higher reduction in antiarrhythmic drug usage postablation after GPA than after PVI (55.5% vs 36%; *P* = .05). Patients were referred for redo ablation procedures in 31% (16 of 52) after GPA and 24% (12 of 50) after PVI (*P* = .53).

**Conclusion:**

GPA did not prevent atrial arrhythmias more than PVI. However, less radiofrequency ablation was delivered to achieve a higher reduction in antiarrhythmic drug usage with GPA than with PVI.

## Introduction

Pulmonary vein isolation (PVI) is the standard ablation technique to treat atrial fibrillation (AF). Randomized trials demonstrate a success rate between 50% and 70% at preventing 30 seconds of AF in patients with paroxysmal AF not controlled by medical therapy.^1^^,^^2^ Strategies for improving this success rate have been confounded by small promising studies of adjunctive ablation targets that were then shown to have a negative impact when tested in large trials such as Substrate and Trigger Ablation for Reduction of Atrial Fibrillation Trial II.^3^ A genuine adjunctive AF target should be effective at preventing AF even without PVI. A strategy of treating the putative AF ablation target alone without PVI and comparing against PVI could be more robust at identifying potential future PVI adjunctive targets. We adopted this approach to study the dense clusters of intrinsic autonomic cardiac nerves that form ganglionated plexuses (GPs) in the epicardium of the atria as potential upstream triggers of AF.

We established in preliminary studies that it was possible to map the entire left atrium for the 2 functional classes of GP: the atrioventricular dissociating GP (AVD-GP), which cause atrioventricular dissociation; and the ectopy-triggering GP (ET-GP), which trigger atrial or pulmonary vein (PV) ectopy and AF.^4^^,^^5^ Subsequently, we performed a randomized controlled single-center pilot study to test the feasibility of locating and ablating GPs using an endocardial approach and showed that selective GP ablation (GPA) was able to prevent AF without the need for PVI.^6^ We made modifications to this pilot protocol and tested the hypothesis that selective GPA could be superior to PVI in a larger, multicenter, randomized controlled setting.

## Methods

GANGLIA-AF (ClinicalTrials.gov identifier NCT02487654) was a prospective, multicenter, randomized, single-blinded clinical study recruiting patients with paroxysmal AF indicated clinically for AF ablation. The recruitment period was between May 2017 and May 2019. Three UK centers participated in the study: Hammersmith Hospital, Imperial College Healthcare NHS Trust, UK; Derriford Hospital, University Hospitals Plymouth NHS Trust, UK; St Bartholomew’s Hospital, Barts Health NHS Trust, UK. All patients gave written informed consent, and the study was approved by the local research ethics committee. Patients with symptomatic drug-refractory paroxysmal AF indicated for ablation were recruited to the study. The inclusion and exclusion criteria are summarized in Online Supplemental Table 1.

Patients were randomized to PVI or to GPA without PVI. Randomization was performed using a permuted block “sealed envelope” approach. The first block of randomization was performed on 21 consecutive patients, randomized 2:1 to GPA (GPA, n = 14; PVI, n = 7). This was in anticipation for a high rate of crossover from the GPA group as experienced in our previous pilot study.^6^ Afterward, randomization was switched to 1:1 in blocks of 10 for the remainder of the recruitment. Patients and their cardiologists providing their usual care were blinded to their randomization. Operators on the day of ablation, the data collector, and the analyst were unblinded to randomization.

### Protocol for all patients

#### Preoperative work-up

All patients discontinued AADs and β-blockers 5 half-lives before the procedure. Amiodarone was discontinued at least 2 months before the procedure. All patients provided written informed consent for participation in the study, which was approved by the health research authority and the local research ethics committee. Complete blood count, urea and electrolytes, C-reactive protein were measured on the morning before the procedure to ensure suitability for ablation.

#### Clinical procedures

All patients had general anesthesia. Local policies were followed for anticoagulation and assessment of cardiac thrombus using transesophageal echocardiography. A decapolar catheter was positioned in the coronary sinus. After transseptal puncture, a 20-pole circumferential catheter (LassoNav, Biosense Webster, Diamond Bar, CA) was used to create a respiratory-gated 3-dimensional electroanatomic map of the left atrium (CARTO, Biosense Webster). Blood pressure was continuously monitored using a radial arterial line. Heparin was administered throughout the cases to maintain the activated clotting time of >300 seconds. All data were recorded at 1000 Hz by the electrophysiology recording system (Bard EP, Lowell, MA). At the end of the procedure, heparin was reversed with protamine, and patients were discharged the next morning if well.

#### GP mapping and ablation

High frequency stimulation (HFS) was performed to locate the left atrial GPs in patients randomized to GPA. A Grass S88 stimulator (Astro-Med, West Warwick, RI) was used to deliver HFS from the distal electrode of a bipolar 3.5-mm irrigated-tip contact force sensing ablation catheter (SmartTouc, Biosense Webster), with a minimum contact force of 3g. A PV catheter was always placed in the nearest PV to the site of HFS testing to capture the earliest PV ectopy signals. Approximately 6 mm spacing was used between each HFS test site to achieve an evenly spaced global left atrial GP map. We did not test for GPs inside the PVs or in the left atrial appendage. We aimed to test up to 80 HFS sites, as this was usually sufficient to cover all the left atrial surface available for testing as in our previous pilot study.^6^ The exact number of HFS sites tested varied between patients, depending on the size of their left atrium and the procedure time.

Two types of HFS techniques were used: “synchronized HFS” to detect ET-GPs and “continuous HFS” to detect AVD-GPs. The synchronized HFS technique was first described by Schauerte et al^7^ in canines, who demonstrated that atrial ectopy–triggering response with HFS are abolished with atropine and attenuated with β-blockade, confirming its autonomic mechanism. This was also reproduced in clinical studies.^6^^,^^8^^,^^9^ In this study, for our synchronized HFS protocol, the left atrium was paced at high output (10 V) from the ablation catheter during sinus rhythm (SR) to check for atrial capture and to exclude ventricular capture. Then, we delivered short bursts of HFS trains (10 V, 80 ms duration, 40 Hz) coupled to each paced stimulus to ensure that HFS was delivered within the local atrial refractory period. This stimulated only the nerves and avoided direct myocardial capture. We delivered up to 15 trains of synchronized HFS per test site. If atrial ectopy or AF was triggered, we discontinued pacing and HFS immediately to avoid direct myocardial capture and sustained AF.

Continuous HFS was performed during AF by delivering HFS (10 V, 20 Hz) continuously for up to 10 seconds or until asystole occurred.

The definition for a *positive ET-GP* was reproducible induction of PV or atrial ectopy, AF, or atrial tachycardia (AT). Reproducibility was assessed up to 3 times with HFS. The definition for a *positive AVD-GP* was ≥50% increase in the R-R interval during HFS compared with the average 10 R-R intervals before HFS.

The main goal of our study was to map ET-GPs with synchronized HFS during SR. However, if patients developed sustained AF during GP mapping, GPs were ablated until successful restoration of SR. A positive response to GPA during sustained AF was termed “acute AF modulation,” and this was defined as follows: (1) termination to SR, (2) organization to AT, and (3) slowing of AF cycle length by ≥30 ms. A subgroup analysis was performed in patients who underwent GPA to correlate long-term freedom from ≥30 seconds of AF/AT recurrence. Patients who underwent GPA were divided into those who had (1) <100% success in acute AF modulation, (2) 100% success in acute AF modulation, and (3) no sustained AF with GP stimulation.

If all GPs were ablated and the patient was still in AF, up to 3 direct current cardioversions were performed to restore SR and to complete GP mapping. If patients remained in incessant AF, AVD-GPs were mapped with continuous HFS and ablated. We targeted AVD-GPs as well in this instance because significant overlap exists with ET-GPs and we wanted to target as many ET-GPs as possible.^10^

After completing GP mapping, all GPs were ablated by delivering a cluster of 3 point-by-point lesions around each GP site, with the contact force of >3g. Each ablation lesion was for 30 seconds at 30 W except in the posterior wall, where it was limited to 25 W. Catheter irrigation was set to 17 mL/min. This was usually adequate to render any GP negative for any response with repeat HFS testing. All ablated GPs were retested with HFS after ablation. If there was still a positive response, further ablation was performed until no further positive responses could be evoked.

### PVI

For patients randomized to the PVI arm, wide antral circumferential ablation was performed around the PVs by using contiguous point-by-point radiofrequency (RF) ablation lesions. The entry and exit blocks of PVs were confirmed using a PV catheter. Operators used the ablation index to guide PVI on >60% of patients who completed PVI.

### Clinical follow-up

Patients were followed up and monitored for 12 months, with continuous remote contact with a clinician. Patients were fitted with 48-hour Holter monitors at 3-, 6-, 9-, 12-month intervals. If patients experienced symptoms between these 3-monthly time points, they were offered further Holter monitors in an attempt to record their symptomatic arrhythmia. Patients with pacemakers, loop recorder devices, and personal AliveCor Kardia electrocardiogram (ECG) (AliveCor Inc, Mountain View, CA) monitoring devices were interrogated throughout their follow-up. Any significant arrhythmia identified on their interrogation counted toward the primary end point regardless of the Holter results. We encouraged patients to discontinue all antiarrhythmic drugs (AADs) following the 3-month blanking period after their ablation procedures, but this was not mandated in the protocol.

### Repeat AF ablation

Patients who were randomized to GPA returning for redo AF ablation procedures had repeat HFS testing. This identified any recurrence of GPs or new GPs not previously identified. All GPs identified in the redo procedure were ablated in addition to PVI. Patients who were randomized to PVI returning for redo AF ablation procedures had repeat PVI only.

### End point definition

The primary end point was any documented atrial arrhythmia (AF, AT, or atrial flutter) lasting for ≥30 seconds consecutively recorded on the Holter monitor, 12-lead ECG, pacemaker, loop recorder, and AliveCor Kardia recorded ECG after a 90-day blanking period.

The secondary end points included repeat ablation for AF/AT/atrial flutter after a 90-day blanking period, mortality, and any significant complications related to the procedure (bleeding, thrombosis, phrenic nerve palsy, and cardiac tamponade) requiring intervention.

### Statistics

Data analyses were conducted using the per-protocol (PP) study population. The PP study population excluded patients who were withdrawn after randomization. For sample size calculation, it was estimated that at a statistical power of 80% at a 5% significance level, 108 patients were required to detect 25% difference in primary end point, where 45% in PVI and 20% in GPA are predicted to have recurrent AF/AT. We enrolled 116 patients to allow for ∼5% not completing follow-up.

Statistical analysis was performed using SPSS Version 28 (IBM Corporation, Armonk, NY) and Prism 5 (GraphPad, San Diego, CA). Continuous variables were expressed as mean ± SD. Categorical variables were expressed as number and percentage. D’Agostino and Pearson omnibus and Shapiro-Wilk tests were performed to assess the normality of continuous variables before the comparison of means tests. Mann-Whitney *U* test, Fisher exact test, and unpaired *t* test were used for comparison of means. An event-free survival was estimated using a Kaplan-Meier curve for the primary end point. Clinical parameters associated with AF recurrence and other procedural parameters were studied using univariate and stepwise multivariate analysis in a Cox regression model. All variables with *P* values ≤.05 from the univariate analysis and well-established risk factors for AF recurrence, such as age and hypertension, were entered into the multivariate regression analysis. A *P* value of <.05 indicated statistical significance.

## Results

**Figure 1:**
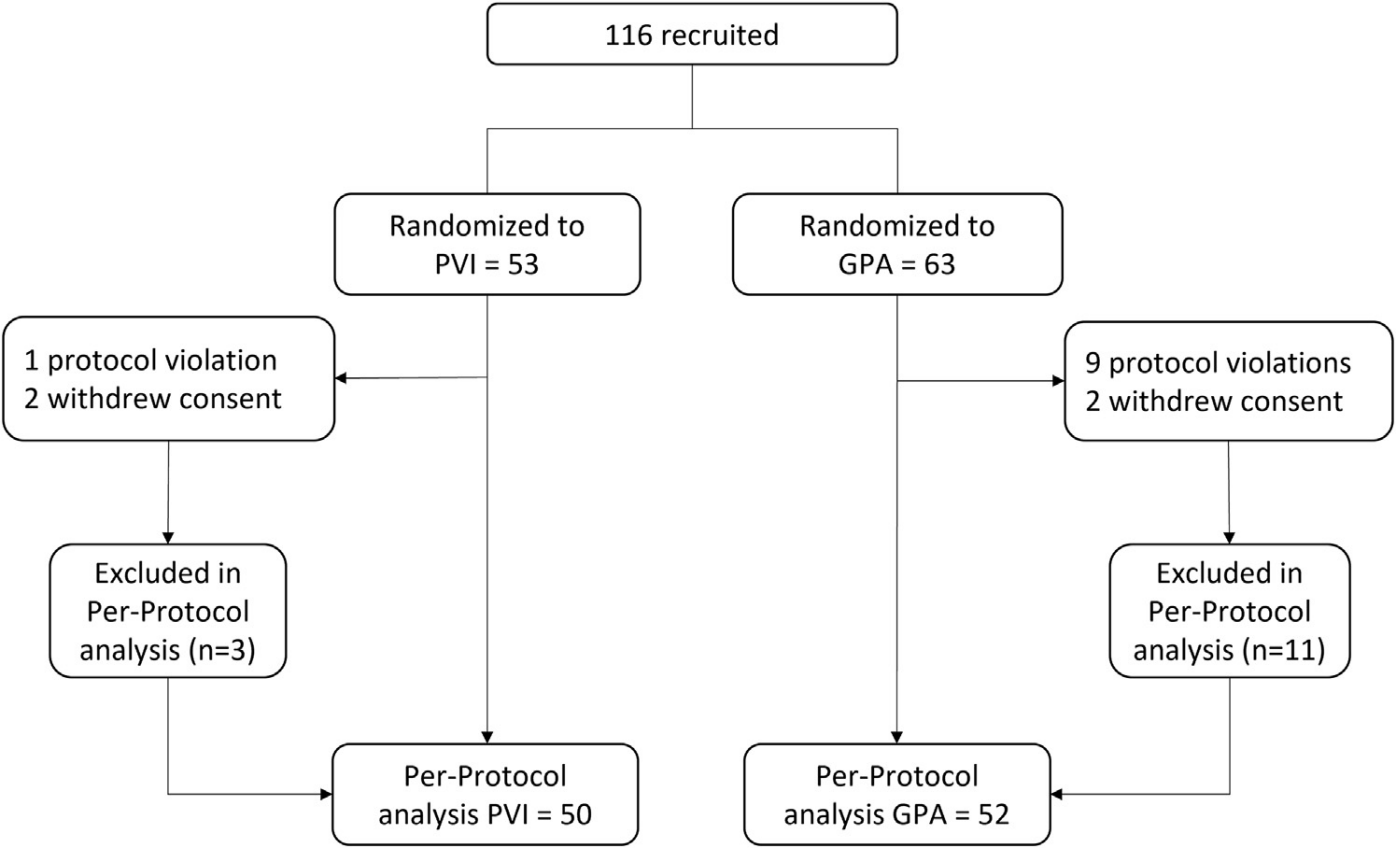
Study flowchart. GPA = ganglionated plexuses ablation; PVI = pulmonary vein isolation.

One hundred sixteen patients were recruited and randomized. Sixty-three patients were randomized to GPA and 53 to PVI (Figure 1). Fourteen patients were withdrawn after randomization (11 from the GPA group; 3 from the PVI group) owing to protocol violations (n = 10) and patient withdrawals (n = 4). Therefore, 102 patients (52 in the GPA group; 50 in the PVI group) were followed up in the PP population for the final analysis. Patients were 63 ± 11 years of age; 70% were men. Patients randomized to PVI had a significantly higher number of patients with a CHA_2_DS_2_-VASc (congestive heart failure, hypertension, age ≥ 75 years, diabetes mellitus, previous stroke, transient ischemic attack, or thromboembolism, vascular disease, age 65–74 years, sex category [female]) score of 1 (50% vs 27%; *P* = .01), and patients randomized to GPA had a significantly higher number of patients on AADs (67% vs 42%; *P* = .02). There was no other significant demographic difference between the 2 groups (Table 1).

**Table 1:** Demographic characteristics of study patients in the per-protocol group

Characteristic	GPA (n = 52)	PVI (n = 50)	*P*
Age (y)	64 ± 11	62 ± 11	.27
Male	35 (67)	36 (72)	.67
BMI (kg/m^2^)	27.0 ± 3.9	28.4 ± 4.9	.13
LA diameter (mm)	3.7 ± 0.6	3.9 ± 0.5	.18
LVEF (%)	63 ± 5	64 ± 5	.33
CHA_2_DS_2_-VASc score			
0	15 (29)	11 (22)	.50
1	13 (27)	25 (50)	.01
2	11 (19)	5 (10)	.17
≥3	13 (31)	8 (16)	.33
HTN	16 (31)	17 (34)	.83
IHD	5 (10)	3 (6)	.72
T2DM	2 (4)	3 (6)	.68
Stroke/TIA/embolus	5 (10)	1 (2)	.21
Sleep apnea	1 (2)	4 (8)	.20
Antiarrhythmic drug	35 (67)	21 (42)	.02
Autonomic symptom triggers	10 (19)	7 (14)	.60

Values are presented as mean ± SD or n (%).

BMI = body mass index; CHA_2_DS_2_-VASc = congestive heart failure, hypertension, age ≥ 75 y, diabetes mellitus, previous stroke, transient ischemic attack, or thromboembolism, vascular disease, age 65–74 y, sex category (female); LA = left atrial; LVEF = left ventricular ejection fraction; GPA = ganglionated plexuses ablation; HTN = hypertension; IHD = ischemic heart disease; PVI = pulmonary vein isolation; T2DM = type 2 diabetes mellitus; TIA = transient ischemic attack.

### Procedure details

**Figure 2:**
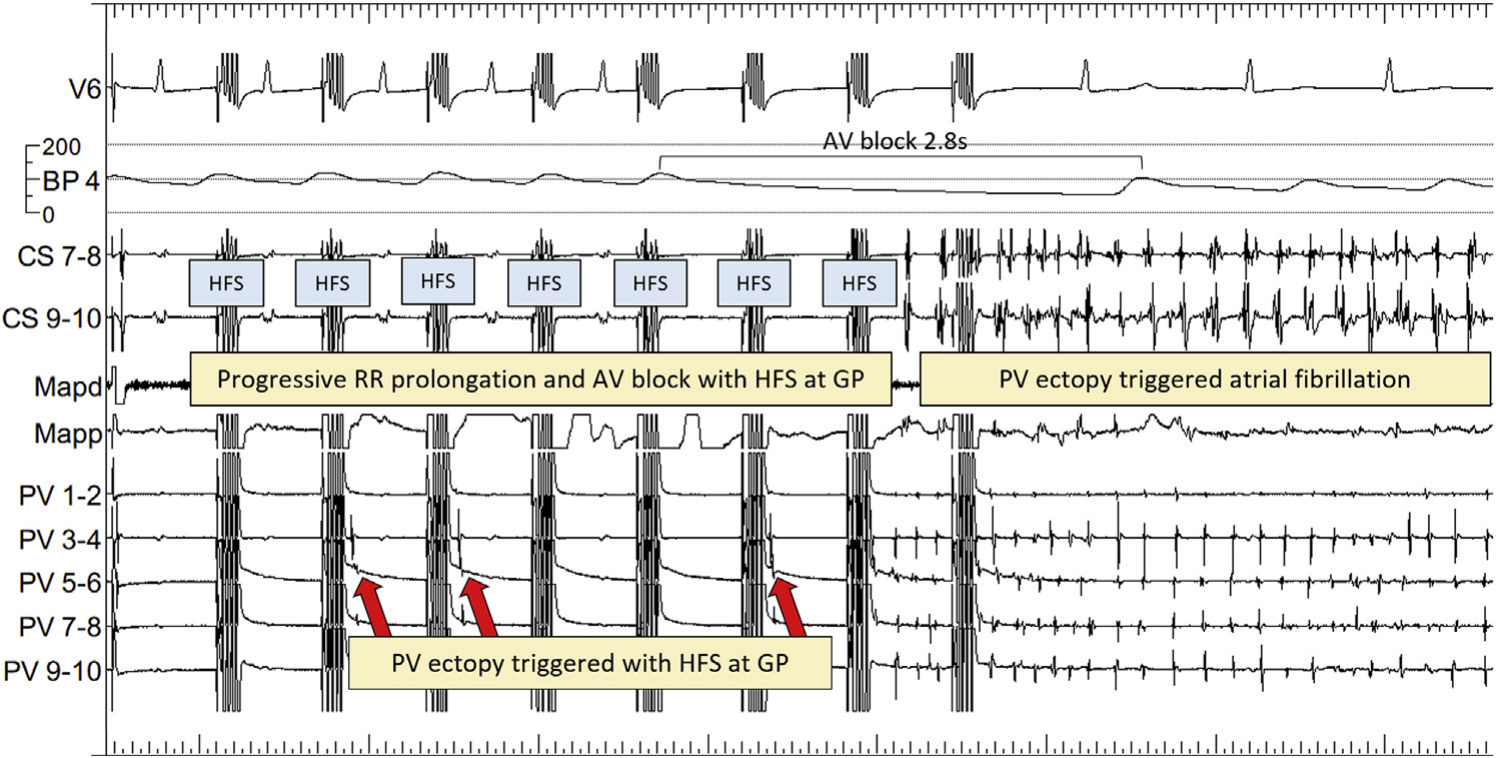
Example of HFS at a GP site. After the second HFS, PV ectopy is triggered (*arrow*) with the earliest signal in PV 3-4. This is repeated with further trains of synchronized HFS. Simultaneously, there is a progressive R-R prolongation until 2.8 seconds of AV block, followed by AF. This GP site demonstrates colocation of ectopy-triggering GP and AV dissociating GP. AF = atrial fibrillation; AV = atrioventricular; BP = blood pressure; CS = coronary sinus; GP = ganglionated plexuses; HFS = high frequency stimulation; Mapd = mapping catheter distal; Mapp = mapping catheter proximal; PV = pulmonary vein.

Patients randomized to GPA had on average 89 ± 26 HFS sites tested per patient, identifying a median of 18.5% (interquartile range 16%–21%) of GPs. In total, 4646 HFS sites were tested and 858 (18.5%) were ET-GPs. Thirty-eight patients (73%) maintained SR throughout the procedure and had ET-GPs mapped and ablated. An example of synchronized HFS triggering PV ectopy followed by AF at an ET-GP site is shown in Figure 2. This particular site also had significant atrioventricular dissociation and asystole with synchronized HFS, identifying this site as both ET-GP and AVD-GP. Further examples of other types of ET-GP responses to HFS, such as triggering single atrial ectopy to short runs of atrial ectopy, can be seen in our previous work.^5^

Fourteen patients (27%) had recurrent sustained AF during synchronized HFS mapping and required further continuous HFS mapping to identify AVD-GPs. This included 6 patients with a mixture of ET-GP and AVD-GPA, 5 patients with only ET-GPA, and 3 patients with only AVD-GPA. The anatomical distribution of GP was categorized according to the left atrial GP atlases we previously described.^4^^,^^5^ ET-GPs had an anatomical distribution different from AVD-GPs: ≥ 20% of the probability distribution of ET-GPs were down the midline of the anterior wall, inferior to the left atrial appendage, all around the left PVs except for the posterior portion of the right inferior PV, across the roof, and left side of the posterior wall; ≥ 20% probability distribution of AVD-GPs were in the anterior portion of the right superior PV, septum, posterior portion of the right inferior PV, inferior border of the left posterior wall, and the left atrial floor.

On average, 73% ± 17% of GPs mapped per patient were within the ≥20% probability distribution areas of the GP atlas, and 27% ± 17% of GPs mapped per patient were within the <20% probability distribution areas of the GP atlas.

All patients randomized to PVI had successful complete PVI, confirmed by demonstration of entry and exit blocks at PVs. The duration of the procedure was 181.3 ± 33.5 minutes in GPA and 127.5 ± 33.2 minutes in PVI (*P* < .0001). The duration of fluoroscopy was 18.3 ± 13.2 minutes in GPA and 12.8 ± 6.9 minutes in PVI (*P* = .11). The total RF ablation time was 22.9 ± 9.8 minutes in GPA and 38 ± 14.4 minutes in PVI (*P* < .0001). The total RF energy used was 35.4 ± 15.6 kWs in GPA and 63.2 ± 23.4 kWs in PVI (*P* < .0001) (Table 2). All patients were discharged the following day without an extended hospital stay.

**Table 2:** Procedure details

Variable	GPA (n = 52)	PVI (n = 50)	*P*
Procedure time (min)	181.3 ± 33.5	127.5 ± 33.2	<.0001
Fluoroscopy time (min)	18.3 ± 13.2	12.8 ± 6.9	.11
RF time (min)	22.9 ± 9.8	38.0 ± 14.4	<.0001
RF energy (kWs)	35.4 ± 15.6	63.2 ± 23.4	<.0001

Values are presented as mean ± SD.

GPA = ganglionated plexuses ablation; PVI = pulmonary vein isolation; RF = radiofrequency.

### Primary end points

**Figure 3:**
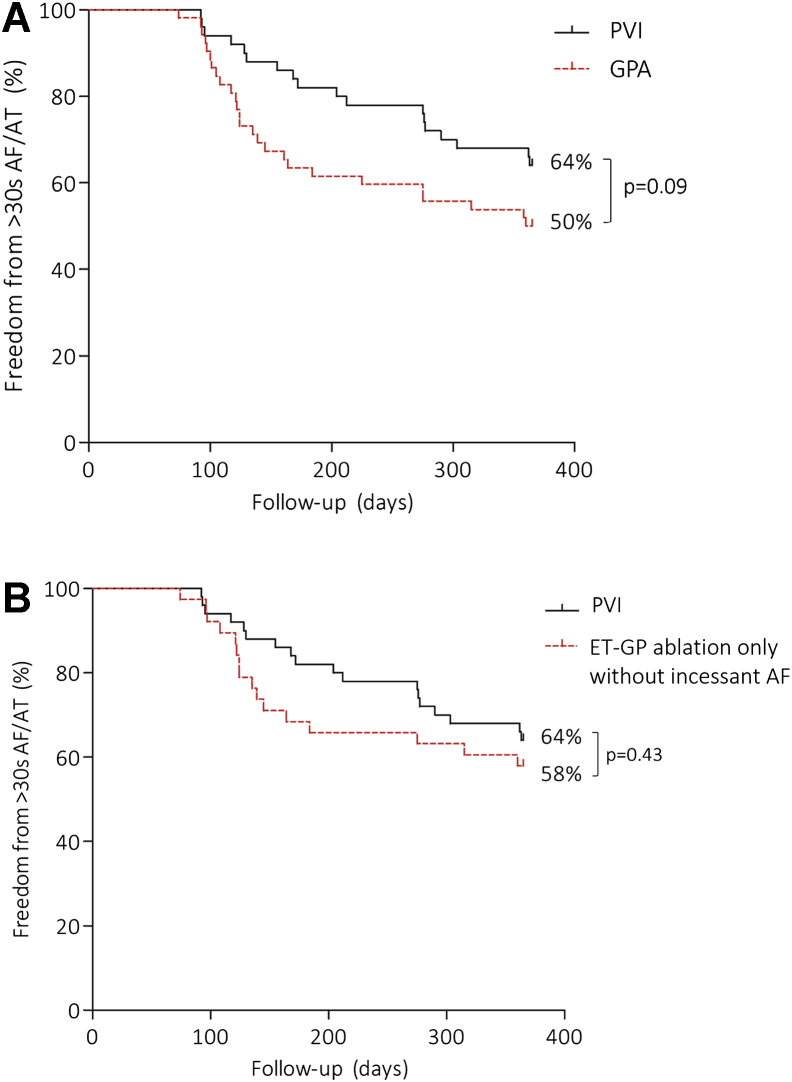
Primary end point. **A:** The primary end points at 12 months of follow-up in the PVI and the overall GPA group were not significantly different from each other. **B:** The GPA subgroup that had undergone ET-GP ablation only without incessant AF during GP mapping had greater freedom from >30 seconds of AF/AT than did the overall GPA group in panel A. AF = atrial fibrillation; AT = atrial tachycardia; ET-GP = ectopy-triggering ganglionated plexuses; GPA = ganglionated plexuses ablation; PVI = pulmonary vein isolation.

**Figure 4:**
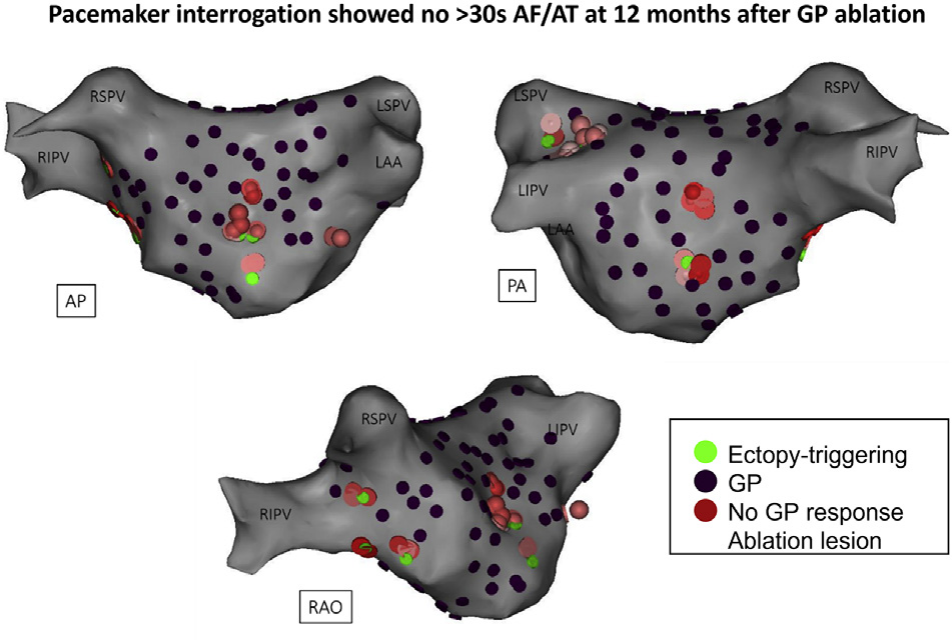
Example of successful ET-GP ablation without PVI. One hundred thirty-five sites were tested with synchronized HFS; 16.5 minutes of RF ablation was performed on 13 ET-GPs. PVs remained electrically connected. Pacemaker interrogation after 12 months showed no evidence of atrial arrhythmia since ET-GP ablation. The patient has remained symptom free and off all antiarrhythmic drugs. AP = anterior posterior; AF = atrial fibrillation; AT = atrial tachycardia; ET-GP = ectopy-triggering ganglionated plexuses; GP = ganglionated plexuses; HFS = high frequency stimulation; LAA = left atrial appendage; LIPV = left inferior pulmonary vein; LSPV = left superior pulmonary vein; PA = posterior anterior; PV = pulmonary vein; RAO = right anterior oblique; RF = radiofrequency; RIPV = right inferior pulmonary vein; RSPV = right superior pulmonary vein.

Twenty-six of 52 (50%) in GPA and 32 of 50 (64%) in PVI were free from the primary end point (log-rank, *P* = .09) (Figure 3A). Specifically, ET-GPA alone without patients requiring continuous HFS mapping owing to incessant AF showed 22 of 38 (58%) free from the primary end point (Figure 3B). An example of successful GPA is shown in Figure 4. This patient had 135 sites tested with synchronized HFS; 16.5 minutes of RF ablation was performed at 13 ET-GPs. PVs remained electrically connected. The patient had a permanent pacemaker interrogated after 12 months of follow-up, which showed no evidence of AF/AT.

Among the patients who reached the primary end point, 5 of 26 (19%) in GPA and 5 of 18 (28%) in PVI had atrial flutter or AT as the primary end point rhythm (*P* = .73).

### Secondary end points

Within the 12-month follow-up period, 16 patients (31%) from the GPA group and 12 patients (24%) from the PVI group were referred for redo AF ablation procedures because of symptomatic recurrence of AF (*P* = .51). Furthermore, there was a significantly higher reduction in the usage of AADs after GPA than after PVI (55.5% vs 36%; *P* = .05).

In the GPA group, 1 patient had a periprocedural complication: a cardiac tamponade during GPA, which resolved with pericardiocentesis. The patient was discharged the following day. There were no major complications with PVI. Interestingly, 7 patients with GPA were hospitalized with symptoms consistent with pericarditis as compared with 1 patient with PVI (*P* = .06). Patients with pericarditis were conservatively managed with a short course of simple analgesia and had no further complications. There was no significant difference in the quantity of ablation in patients with pericarditis compared with those without, but in patients randomized to GPA, there was a predilection of GPA down the midline of the left atrial anterior wall.

### Follow-up ECG monitoring and AADs

Patients who did not reach the primary end point had on average 3 ± 1 48-hour Holter monitors fitted per patient. There was no difference in the number of Holter monitors fitted between the 2 groups (*P* = 1.00). Twelve patients had additional ECGs to Holter monitors, including 9 patients with AliveCor ECG recorders, 3 patients with permanent pacemaker devices, and 1 patient with an implanted loop recorder. Eight of these patients (12%) were randomized to GPA and 4 (7%) to PVI.

From the Holter ECG analysis, the GPA group had an increased mean heart rate (HR) at 12 months compared with preablation (70 ± 12 beats/min vs 64 ± 9 beats/min; *P* = .06) and the PVI group had an increased mean HR at 6 months compared with preablation (71 ± 11 beats/min vs 67 ± 15 beats/min; *P* = .06) but this was not statistically significant. In the heart rate variability (HRV) analysis, the standard deviation of R-R intervals over the entire analyzed period at 12 months was significantly higher in GPA than in PVI (137 ± 30 ms vs 102 ± 24 ms; *P* = .03). There were no other significant differences in HRV parameters during follow-up.

At the index procedure, more patients were on AADs in the GPA group (35 of 52 [67%]) than in the PVI group (21 of 50 [42%]; *P* = .02). Asymptomatic patients postablation were encouraged to discontinue AADs after the blanking period, but this was not mandated by the protocol. During follow-up after the blanking period, 6 of 52 (11.5%) post-GPA and 3 of 50 (6%) post-PVI (*P* = .49) remained on AADs. Compared with baseline, GPA led to a significantly larger reduction of patients using AADs postablation than did PVI (55.5% reduction vs 36% reduction; *P* = .05).

### Acute and long-term AF modulation with GPA

**Figure 5:**
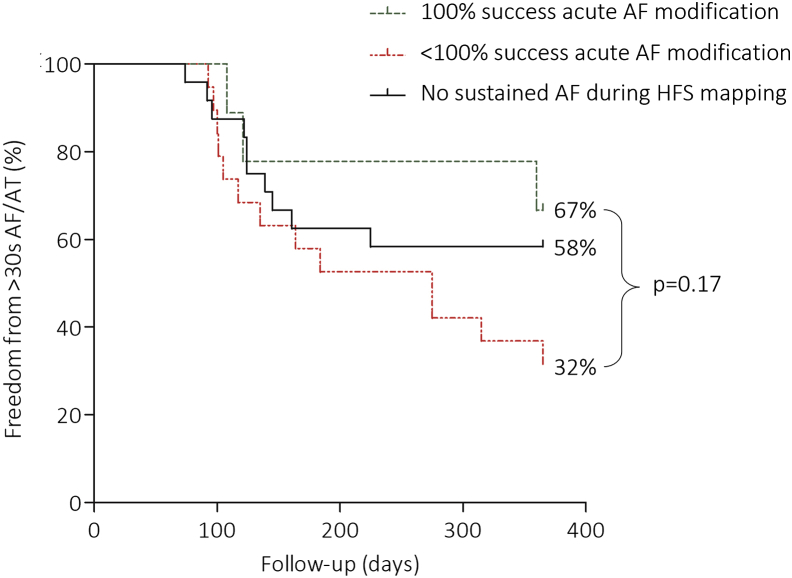
Acute AF modification with GPA and long-term outcomes. If >2 minutes of sustained AF was triggered during HFS mapping, GPA was attempted to try and restore SR. Acute AF modification included AF termination to SR, organization to AT, or prolongation of AF cycle length by ≥30 ms. There was a range of success in acute AF modification between patients, which was divided into 100% success in acute AF modification and <100% success in acute AF modification. These subgroups were also compared with patients who did not have sustained AF during HFS mapping. The differences in long-term freedom from ≥30 seconds of AF/AT after GPA in these subgroups was not statistically significant. SR = sinus rhythm; other abbreviations as in Figure 3.

In a further subgroup analysis of patients who had undergone GPA, 28 (54%) had ≥2 minutes of sustained AF during HFS mapping. There were 77 attempts at ablating GPs to try and restore SR in these patients. Fifty-six percent of the attempts (43 of 77) were successful at acutely modulating AF, and a range of responses was observed: 79% (34 of 43) terminated to SR, 14% (6 of 43) organized to AT, and 7% (3 of 43) had increased AF cycle length by ≥30 ms. There were on average 2 ± 1 GPA attempts per patient during sustained AF. Patients’ overall success at acutely modulating AF with GPA was correlated with their primary end points at 12 months of follow-up. Of the 9 patients who had 100% success in acute AF modulation, 6 (67%) were free from ≥30 seconds of AF/AT. Of the 19 patients who had <100% success in acute AF modulation, 6 (32%) were free from ≥30 seconds of AF/AT. Also, of the 24 patients who did not have any sustained AF during HFS mapping, 14 (58%) were free from ≥30 seconds of AF/AT. The estimated event-free survival differences between these subgroups were not statistically significant (log-rank, *P* = .17) (Figure 5).

### Predictors of AF/AT recurrence

**Figure 6:**
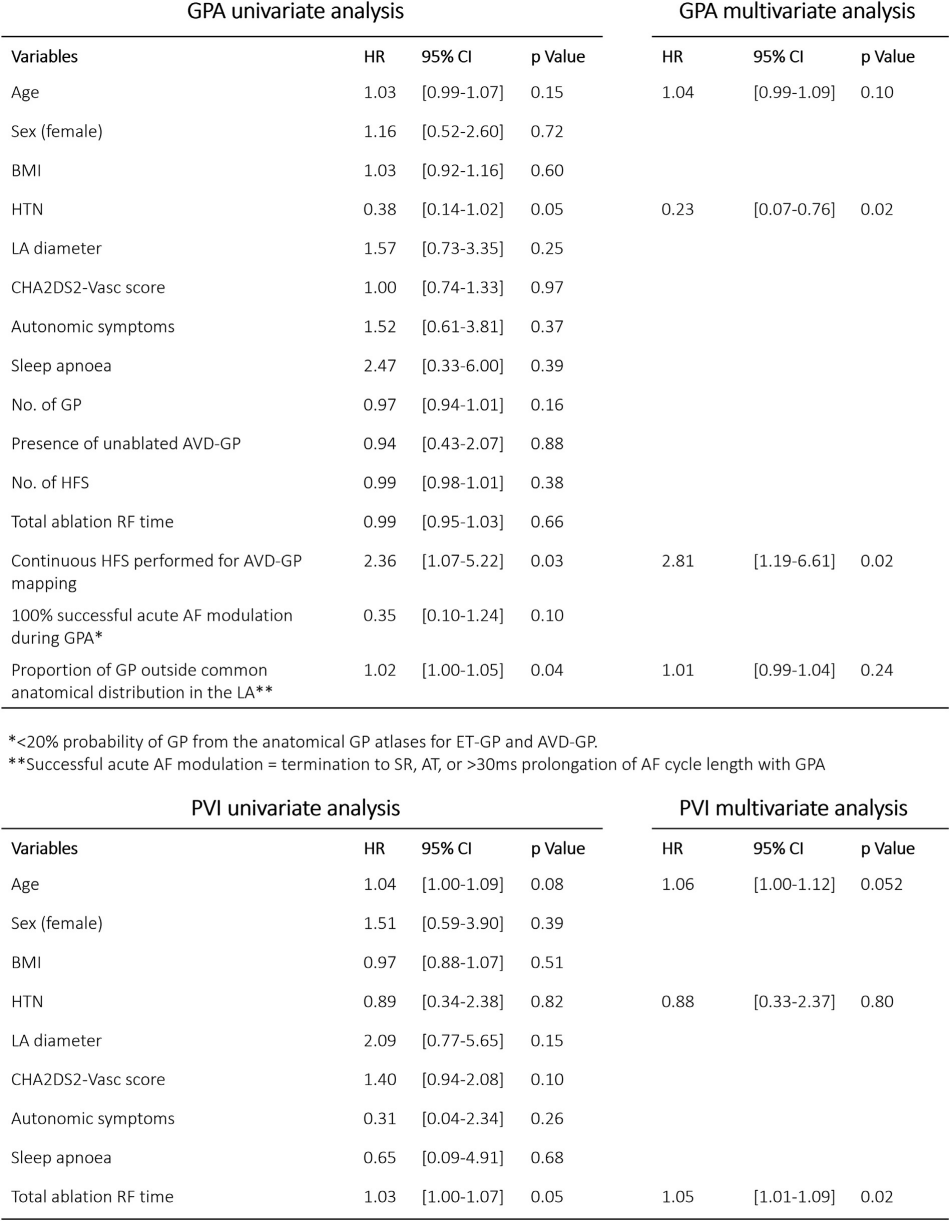
Univariate and multivariate analyses in GPA and PVI groups. All variables with *P* values ≤0.05 from the univariate analysis and well-established risk factors for AF recurrence, such as age and hypertension, were entered into the multivariate regression analysis. AF = atrial fibrillation; AT = atrial tachycardia; AVD-GP = atrioventricular dissociating ganglionated plexuses; BMI = body mass index; CHA_2_DS_2_-VASc = congestive heart failure, hypertension, age ≥ 75 years, diabetes mellitus, previous stroke, transient ischemic attack, or thromboembolism, vascular disease, age 65–74 years, sex category (female); CI = confidence interval; ET-GP = ectopy-triggering ganglionated plexuses; GP = ganglionated plexuses; GPA = ganglionated plexuses ablation; HFS = high frequency stimulation; HR = hazard ratio; HTN = hypertension; LA = left atrial; PVI = pulmonary vein isolation; SR = sinus rhythm; RF = radiofrequency.

Univariate and multivariate analyses were performed on AF/AT recurrence with GPA (Figure 6). In multivariate analyses, having a background of hypertension (hazard ratio 0.23; 95% confidence interval 0.07–0.76; *P* = .02) and incessant AF despite ablating ET-GPs (which then required continuous HFS mapping for AVD-GPs) (hazard ratio 2.81; 95% confidence interval 1.19–6.61; *P* = .02) were significant independent determinants of AF/AT recurrence after GPA. Removing the patients of the latter group (n = 14) from the primary end point analysis in GPA resulted in 22 of 38 (58%) patients with ET-GPA only who were free from AF/AT at 12 months of follow-up (Figure 3B).

Univariate and multivariate analyses were also performed in the PVI group. The total RF ablation time (hazard ratio 1.05 per 1-minute increase; 95% confidence interval 1.01–1.09; *P* = .02) was the only significant independent determinant of AF/AT recurrence.

## Discussion

This study showed that GPA did not prevent atrial arrhythmias more than PVI, and GPA had a significantly longer procedure time, primarily because of the HFS mapping time. However, with GPA, there was significantly less RF ablation and a higher reduction in usage of AADs postablation than with PVI. Interestingly, GPA was particularly effective in patients with hypertension, which has been a marker of failure in previous AF ablation studies with circumferential PVI.^11^ The success rate of GPA was slightly improved from our previous pilot study, which also primarily targeted ET-GPs without PVI.^6^ Our success rate for PVI was similar to those reported in large trials of RF ablation for paroxysmal AF.^1^^,^^2^

Previously, selective AVD-GPA alone was shown to prevent symptomatic paroxysmal AF and improved symptoms in 42.5% among 40 patients.^12^ Selective ablation has generally been considered as too time-consuming, and empirical ablation of anatomical sites for GPs was proposed as an adjunct to PVI. This was found to be superior to both empirical GPA alone and PVI alone in a randomized controlled trial.^13^ However, a study of thoracoscopic AF ablation with adjunctive epicardial GPA did not show the same benefit.^14^ Our study is the first to investigate the effects of ablating ET-GPs alone compared with PVI. We were able to achieve a 58% success rate in the subgroup that had undergone ET-GPA exclusively compared with the 50% success rate in the overall GPA group, which included AVD-GPA procedures. The majority of ET-GP were around the 4 PVs, across the midline of the roof, down the mid-anterior wall, just inferior to the left atrial appendage, and the left side of the posterior wall. The majority of AVD-GPs were in the inferior portion of the posterior wall and the floor. These variations in the anatomical distribution highlight the importance of using functional mapping with HFS to differentiate GPs.

Patients who underwent GPA requiring continuous HFS mapping because of incessant AF were 2.8 times more likely to have AF/AT recurrence. It is not clear whether this is because patients who developed incessant AF had a more advanced substrate or AVD-GPs were less specific to the pathogenesis of AF. It was also noted that 100% acute modification of sustained AF by GPA was associated with greater freedom from atrial arrhythmia recurrence, but this was not statistically significant. Methods to reduce the requirement for continuous HFS due to sustained AF and maintain SR need further exploration.

HRV and HR changes have been reported in the past as an indicator for autonomic remodeling after GPA.^12^^,^^15^ In our study, the standard deviation of RR intervals over the entire analyzed period was the only significant HRV change 12 months after GPA compared with baseline. In another study, the standard deviation of RR intervals were significantly increased in patients who had AF recurrence compared to those without AF, up to 6 months after GPA.^12^ However, we found no such correlation within our GPA group at any time point. The mean HR increase was more durable with GPA vs baseline at 12 months as compared with 6 months with PVI. This may suggest more permanent autonomic remodeling after GPA, but with no association with AF/AT recurrences.

Hypertension plays an important role in AF pathogenesis,^16^ associated with increased left atrial size^17^ and fibrosis leading to more arrhythmogenic substrates. Its role in success of AF ablation is less clear.^18^ Interestingly, our multivariate analyses showed that having hypertension improved the likelihood of success with GPA, significantly reducing the rate of AF/AT by 77%, compared to those without hypertension. Dysfunctional autonomic nervous system, particularly the sympathetic division, has been associated with hypertension.^19^ It is possible that AF was sympathetically driven in patients with hypertension and were more susceptible to GPA.

### Study limitations

This is the first clinical study to map and target ET-GPs in patients. Reproducibility of our findings by other centers need to be demonstrated, and therefore, ET-GPA in AF remains investigational. It is important to note that our finding of a median of 18.5 GP sites per patient relates to the finding of GPs as characterized by their electrophysiological responses to HFS, not the whole anatomical distribution of GPs. Four patients withdrawn from the GPA group did not have any identifiable AVD-GP with continuous HFS mapping. This is likely due to inadequate current delivery to the epicardial surface to stimulate the AVD-GP with the Grass S88 stimulator. Therefore, we cannot be sure that other GPs may have been missed because of inadequate stimulation. HFS is the only method that can specifically map and differentiate ET-GPs, but improvements are required in the neurostimulation device to increase sensitivity for ET-GPs identification. Maintaining SR throughout the procedure and possibly patient selection are important determinants for the long-term success of GPA.

## Conclusion

GPA did not prevent atrial arrhythmias more than PVI, and GPA had a longer procedure time, primarily because of the HFS mapping time. However, with GPA, significantly less RF ablation was used to achieve a higher reduction in AAD usage than with PVI. Patients with hypertension appear to be a subgroup that get better outcomes with GPA. Although GPA is currently not an adequate alternative to PVI, improving the technology for locating ET-GPs while maintaining SR and reducing the mapping time may also improve outcomes for GPA.
