# Supplementary material for: Ectopy-triggering ganglionated plexuses ablation to prevent atrial fibrillation: GANGLIA-AF study
Source: Heart Rhythm. 2022 Apr;19(4):516–24. doi: 10.1016/j.hrthm.2021.12.010 (PMC8976158; doi:10.1016/j.hrthm.2021.12.010)
Supplement: Supplemental Table 1 [file mmc1.docx]

**SUPPLEMENTARY MATERIALS**

**For Publication as Data Supplement**

Supplementary Table 1 Inclusion and exclusion criteria of the study.

| **Inclusion criteria** | **Exclusion criteria** |
| --- | --- |
| - Males or females from 18 to 85yrs old - Paroxysmal atrial fibrillation - Off amiodarone for at least 60 days - Suitable candidate for catheter ablation - Signed informed consent | - Contraindication to catheter ablation - Contraindication for general anaesthetic - Presence of a left ventricular thrombus - Previous left atrial ablation - Valvular disease that is grade moderate or greater - Any form of cardiomyopathy - Severe cerebrovascular disease - Active gastrointestinal bleeding - Serum Creatinine >200umol/L or on dialysis or at risk of requiring dialysis - Active infection or fever - Life expectancy shorter than the duration of the trial - Allergy to contrast - Moderate to severe heart failure and/or NYHA Class III-IV - Bleeding or clotting disorders or inability to receive heparin - Uncontrolled diabetes (HbA1c ≥73mmol/mol or HbA1c ≤64mmol/mol and Fasting Blood Glucose ≥9.2mmol/L) - Malignancy needing therapy - Pregnancy or women of childbearing potential not using a highly effective method of contraception - Unable to give informed consent or has insufficient comprehension |

(NYHA=New York Heart Association)
